# Sepsis Secondary to Crusted Scabies Following Iatrogenic Treatment for Hansen’s Disease in an Indigenous Amazonian Patient: A Case Report

**DOI:** 10.1155/crdi/5279255

**Published:** 2026-09-02

**Authors:** Ana Beatriz Nardelli da Silva, Maitê Silva Martins Gadelha, Giovanna Lourenço Cei, Adriani Ferreira do Carmo, Arlisson Macedo Rodrigues

**Affiliations:** ^1^ Department of Life Sciences, University of Bradford, Bradford, UK, bradford.ac.uk; ^2^ Department of Pharmacology and Toxicology of Natural Products, Federal University of Pará, Belém, Pará, Brazil, ufpa.br; ^3^ Department of Public Health, University of Edinburgh, Edinburgh, UK, ed.ac.uk; ^4^ Department of Internal Medicine, João de Barros Barreto University Hospital, Federal University of Pará, Belém, Pará, Brazil, ufpa.br; ^5^ Department of Dermatology, João de Barros Barreto University Hospital, Federal University of Pará, Belém, Pará, Brazil, ufpa.br

**Keywords:** costicosteroids, crusted scabies, Hansen’s disease misdiagnosis, ivermectin, sepsis

## Abstract

**Introduction:**

Sepsis secondary to crusted scabies is a rare but life‐threatening complication of *Sarcoptes scabiei* infestation, particularly in vulnerable and underserved populations. Misdiagnosis with other dermatological conditions may lead to inappropriate treatment and severe outcomes.

**Clinical Findings:**

A 50‐year‐old Indigenous man from the Brazilian Amazon presented with a one‐year history of generalized pruritus, diffuse hyperkeratotic and crusted skin lesions, and progressive clinical deterioration.

**Diagnosis, Interventions, and Outcomes:**

The patient was initially misdiagnosed with Hansen’s disease and atopic dermatitis and received prolonged corticosteroid therapy, which exacerbated his condition. Skin scraping confirmed crusted scabies, and blood cultures grew *Staphylococcus aureus*, establishing the diagnosis of sepsis secondary to skin infection. He was treated with oral ivermectin, topical 5% permethrin, and systemic antibiotics, with gradual tapering and discontinuation of corticosteroids. The patient achieved complete clinical recovery.

**Conclusion (Take‐Home Message):**

This case highlights the critical importance of early recognition of crusted scabies and the risks associated with misdiagnosis and inappropriate immunosuppressive therapy. Prompt diagnosis and treatment are essential to prevent severe complications such as sepsis, particularly in resource‐limited and endemic settings.

## 1. Introduction

Scabies is a highly contagious parasitic dermatosis affecting more than 200 million people worldwide and is strongly associated with poverty, overcrowding, and limited access to healthcare. Crusted scabies is a severe form of *Sarcoptes scabiei* infestation that is commonly associated with immunosuppression, delayed diagnosis, or inappropriate management resulting in extensive mite proliferation, disruption of the skin barrier, and serious systemic complications [1].

Iatrogenic complications related to corticosteroid use represent an important clinical concern, particularly when an underlying infectious condition is misdiagnosed. In tropical and resource‐limited settings, such as the Brazilian Amazon, infectious and parasitic dermatoses, including scabies and Hansen’s disease, remain prevalent and may resemble inflammatory or autoimmune skin disorders [1, 2]. This diagnostic overlap may lead to inappropriate corticosteroid treatment, which can worsen the underlying infection, modify its clinical presentation, and increase the risk of severe complications, including secondary bacterial infection and sepsis, a major cause of morbidity and mortality worldwide [1–3].

Here, we report the case of a 50‐year‐old Indigenous man from the Brazilian Amazon who developed sepsis following prolonged corticosteroid therapy for a misdiagnosed chronic pruritic dermatosis, later confirmed to be crusted scabies. This case highlights the critical importance of accurate differential diagnosis in endemic regions and underscores the risks associated with inappropriate immunosuppressive treatment.

## 2. Case Presentation

A 50‐year‐old Indigenous man from a remote riverside community in the Marajó region, Pará, Brazilian Amazon, presented to the emergency department with signs of systemic infection. He had limited access to healthcare services and fragmented prior medical follow‐up.

At hospital admission, the patient reported a 1‐year history of intense, generalized pruritus that had remained severe throughout the illness. The pruritus persisted despite progressive hyperkeratosis and crust formation and did not meaningfully improve during prior misdiagnosis. At the presentation, he reported fever, malaise, and systemic deterioration. Physical examination revealed widespread hyperkeratotic, crusted, and excoriated lesions associated with purulent discharge. The patient had no known chronic comorbidities and tested negative for HIV, hepatitis B and C, and syphilis. There was no relevant family history of dermatological or immunological diseases. His psychosocial context was notable for residence in an underserved, resource‐limited Amazonian setting with restricted access to specialized care, which contributed to delayed diagnosis and fragmented treatment. No genetic conditions were reported.

**FIGURE 1:**
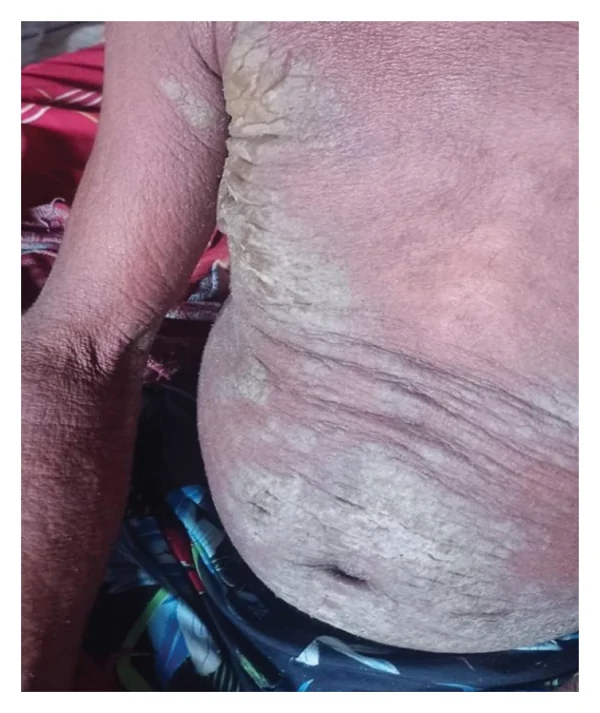
Widespread crusted and scaly lesions on admission with purulent exudate consistent with secondary bacterial infection.

The patient initially received intermittent oral corticosteroid therapy (dexamethasone 4 mg as needed) without a definitive diagnosis, without a definitive diagnosis, resulting in improvement in neither pruritus nor lesions. Subsequently, he was misdiagnosed with Hansen’s disease and treated with multidrug therapy (rifampicin, dapsone, and clofazimine) for 28 days, also without clinical improvement. After referral to a specialized center, bacilloscopy was negative, and treatment for leprosy was discontinued. A presumptive diagnosis of atopic dermatitis was made, and corticosteroid use was continued, leading to progressive worsening of skin lesions and pruritus and eventual development of secondary bacterial infection and sepsis. On admission, the patient was febrile (39°C), hypotensive (90/65 mmHg), and tachycardic (105 beats per minute), with preserved oxygen saturation (98% on room air). He appeared acutely ill, with signs of systemic infection. Dermatological examination revealed extensive hyperkeratotic, crusted, and scaly lesions distributed over the scalp, ear pinnae, trunk, palms, soles, and perineal region. Multiple areas showed excoriations and purulent exudate, consistent with secondary bacterial infection. The skin was diffusely xerotic and thickened, with prominent involvement of intertriginous areas, including axillary, inframammary, inguinal, and periumbilical regions (Figure 1).

**FIGURE 2:**
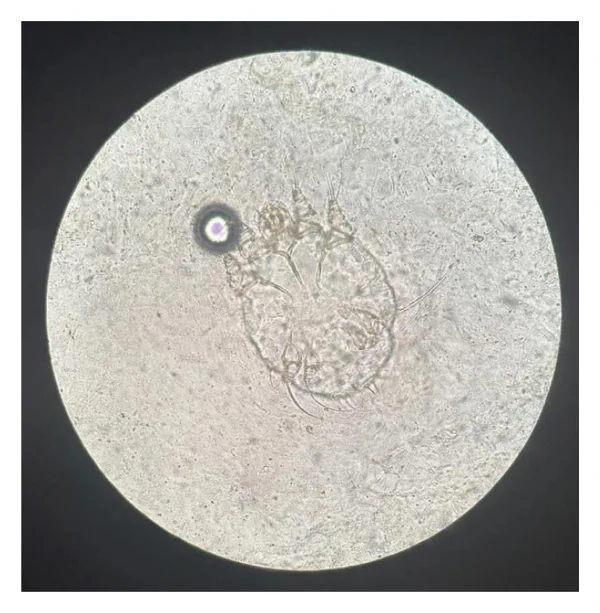
Microscopy of skin scraping showing numerous *Sarcoptes scabiei* mites (arrow), confirming crusted scabies diagnosis.

Laboratory investigations on admission showed a white blood cell count of 9190/μL (reference interval: 4000–11,000/μL), an absolute neutrophil count of 7719/μL (reference interval: 1600–8000/μL), and an elevated C‐reactive protein concentration of 49.6 mg/L (reference value: < 3 mg/L). Repeat testing demonstrated leukocytosis, with a white blood cell count of 12,990/μL (reference interval: 4000–11,000/μL), together with a persistent inflammatory response. Blood cultures were positive for *Staphylococcus aureus*. Serological tests for HIV, syphilis, hepatitis B, and hepatitis C were nonreactive. Microscopic examination of skin scrapings with potassium hydroxide preparation revealed numerous *Sarcoptes scabiei* mites, confirming crusted scabies (Figure 2). Diagnosis was delayed due to limited access to specialized dermatological care in a remote Amazonian setting, as well as overlapping clinical features with other endemic diseases such as Hansen’s disease. Fragmented healthcare access and reliance on empirical treatment contributed to misdiagnosis and inappropriate corticosteroid use. Differential diagnoses included Hansen’s disease and atopic dermatitis, both of which were excluded based on a lack of clinical response and negative bacilloscopy.

Oral ivermectin (∼200 μg/kg per dose, 6 mg) was administered on Days 1, 4, 8, and 9. Topical 5% permethrin was applied on Days 1, 4, 7, 10, and 13. Empirical antibiotic therapy was initiated with oral ciprofloxacin (500 mg every 12 h) and clindamycin (150 mg every 8 h). Because fever and the inflammatory response persisted and blood cultures identified *Staphylococcus aureus* bacteremia, antibiotic therapy was changed to intravenous oxacillin (2 g every 6 h) for 7 days, completing a total antibiotic course of 14 days. Dexamethasone was gradually tapered and discontinued over 21 days to limit further immunosuppression while reducing the risk of adrenal insufficiency.

**FIGURE 3:**
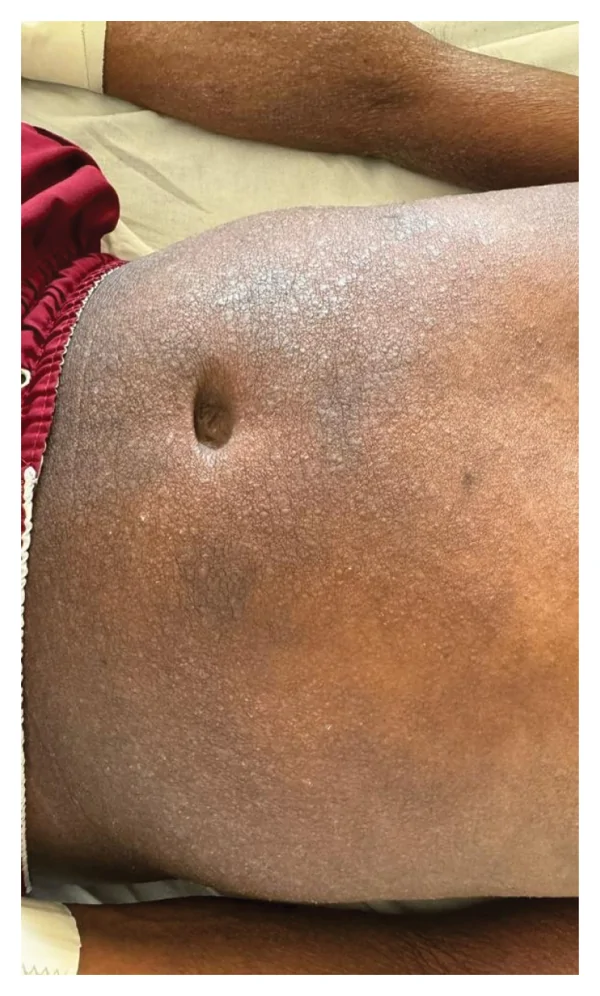
Evolution of skin lesions during treatment with oral ivermectin and 5% topical permethrin.

**FIGURE 4:**
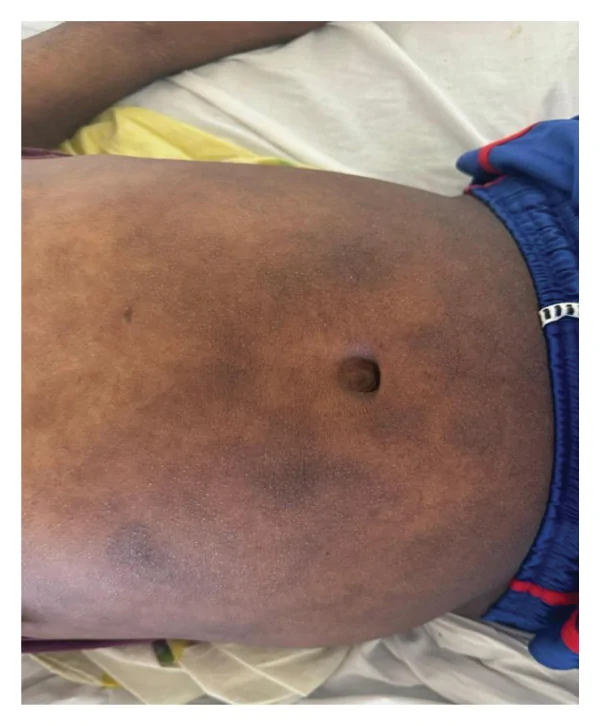
Clinical improvement of skin lesions after 13‐day treatment with oral ivermectin and topical 5% permethrin.

The patient showed marked clinical improvement, with resolution of fever, normalization of vital signs, and significant reduction of pruritus. Skin lesions progressively healed, with near‐complete resolution at discharge (Figures 3 and 4). Follow‐up blood cultures were negative. Inflammatory markers, including C‐reactive protein and erythrocyte sedimentation rate, returned to normal levels prior to discharge. The patient demonstrated good adherence to both systemic and topical treatments during hospitalization, with no reported difficulties in administration. All therapies were well tolerated. No adverse drug reactions or unexpected events were observed during treatment. The clinical course was notable for progressive improvement following appropriate therapy. The patient’s clinical course and therapeutic interventions are summarized in Table 1.

**TABLE 1:** Timeline of the patient’s clinical course and therapeutic interventions.

Period	Clinical events and investigations	Treatment/intervention
Approximately 12 months before admission	Onset of intense generalized pruritus with progressive diffuse skin lesions.	Intermittent oral dexamethasone was used empirically without a definitive diagnosis; no meaningful improvement occurred.
During the subsequent clinical course	The patient was diagnosed clinically with Hansen’s disease despite the absence of definitive microbiological confirmation.	Rifampicin, dapsone, and clofazimine were administered for 28 days; symptoms and lesions did not improve.
After referral to a specialized service	Bacilloscopy was negative. Hansen’s disease treatment was discontinued. Atopic dermatitis was then considered, but the lesions and pruritus continued to worsen.	Corticosteroid exposure continued. The exact regimen and cumulative exposure should be confirmed from the clinical record.
Hospital admission (Day 1)	Fever (39°C), hypotension (90/65 mmHg), tachycardia (105 beats/min), diffuse hyperkeratotic crusted lesions, excoriations, and purulent exudate. Blood cultures grew *Staphylococcus aureus*. Skin scraping demonstrated numerous *Sarcoptes scabiei* mites.	Crusted scabies with secondary bacterial bloodstream infection was diagnosed. Empirical antibiotics and antiparasitic therapy were initiated.
Days 1–13	Progressive improvement of pruritus, fever, inflammatory response, and skin lesions.	Oral ivermectin was administered on Days 1, 4, 8, and 9. Topical 5% permethrin was applied on Days 1, 4, 7, 10, and 13.
During hospitalization	Persistent fever and inflammatory response initially; subsequent blood cultures were negative and inflammatory markers normalized.	Oral ciprofloxacin and clindamycin were changed to intravenous oxacillin after identification of *S. aureus* bacteremia. Total antibiotic duration was 14 days. Dexamethasone was tapered and discontinued over 21 days.
Discharge	Resolution of systemic signs, complete resolution of pruritus, progressive healing of skin lesions, and complete clinical resolution.	No adverse drug reactions were reported. The patient completed the planned treatment course.

*Note:* Day 1 corresponds to the day of hospital admission. IV, intravenous; KOH, potassium hydroxide; MDT, multidrug therapy.

Abbreviations: CRP, C‐reactive protein; ESR, erythrocyte sedimentation rate; *S. aureus*, *Staphylococcus aureus*.

## 3. Discussion

Sepsis resulting from cutaneous infections, particularly when exacerbated by iatrogenic corticosteroid use, represents a severe and potentially life‐threatening condition. Crusted scabies, previously known as Norwegian scabies, is a highly transmissible and severe form of *Sarcoptes scabiei* infestation characterized by extensive mite proliferation, hyperkeratotic crusting, and disruption of the skin barrier [1, 4]. Although it is most commonly associated with immunosuppression, other recognized predisposing factors include diabetes mellitus, advanced age, immobility, neurological or physical impairment, institutional residence, and systemic or potent topical corticosteroid exposure [2, 4].

A systematic review including 683 patients identified corticosteroids as the most frequently reported immunosuppressive trigger, occurring in approximately one‐quarter of cases [5]. However, other relevant risk factors include diabetes mellitus, advanced age, immobility, chronic comorbidities, and impaired cellular immune responses [4]. For example, Mazzola et al. described crusted scabies in an older HIV‐negative patient with diabetes mellitus and prolonged immobility, illustrating that multiple non–HIV‐related factors may contribute to uncontrolled mite proliferation [2]. Although some patients have no identifiable predisposing condition, even inappropriate or relatively limited corticosteroid exposure may modify the clinical presentation, facilitate mite proliferation, and delay the correct diagnosis [6–8].

Crusted scabies may be confused with Hansen’s disease in endemic settings because both conditions can present with chronic and extensive cutaneous lesions [9]. Crusted scabies is typically characterized by diffuse hyperkeratotic, crusted, and scaly plaques, often involving intertriginous regions. In contrast, Hansen’s disease is a dermatoneurological condition that commonly presents with hypopigmented or erythematous lesions associated with sensory impairment and peripheral nerve involvement. Although clinical history and dermatological examination are important in both conditions, the diagnostic investigations differ [9]. In the present case, bacilloscopy was negative, and the patient showed no clinical improvement after 28 days of multidrug therapy for presumed Hansen’s disease. Because negative bacilloscopy alone does not exclude Hansen’s disease, the diagnosis of crusted scabies was established by the identification of numerous *Sarcoptes scabiei* mites on skin scraping, together with the characteristic morphology and distribution of the lesions and the subsequent complete response to antiparasitic treatment.

Previous reports indicate that disruption of the skin barrier in crusted scabies may facilitate bacterial invasion and lead to bacteremia, sepsis, or septic shock. Lin et al. described methicillin‐resistant *Staphylococcus aureus* bacteremia in an 85‐year‐old institutionalized woman with severe dementia and immobility [10]. Although our patient was younger and lived in a remote Amazonian community, both cases involved *Staphylococcus aureus* bloodstream infection. Komiyama et al. reported fatal *Pseudomonas* sepsis in an immunosuppressed patient [11], whereas Wang et al. described fatal septic shock in a 58‐year‐old man with poorly controlled Type 2 diabetes, chronic dermatitis, and multiorgan dysfunction [12]. In the present case, there was no previously documented chronic immunodeficiency or diagnosis of diabetes, although an HbA1c concentration of 8.3% suggested previously unrecognized diabetes or sustained hyperglycemia. Prolonged iatrogenic corticosteroid exposure may also have contributed to disease progression. Unlike the fatal cases previously reported, our patient achieved complete clinical resolution following antiparasitic treatment, systemic antibiotic therapy, and corticosteroid withdrawal.

## 4. Conclusion

Crusted scabies remains a major diagnostic and therapeutic challenge, particularly in underserved and resource‐limited populations. Clinicians should maintain a high index of suspicion in patients with persistent or unexplained pruritus and carefully consider the local epidemiological context before initiating empirical corticosteroid therapy. Delayed or incorrect diagnosis may lead to rapid disease progression and severe complications, including secondary bacterial infection and sepsis. Early recognition, prompt diagnosis, and appropriate antiparasitic treatment are essential to improve outcomes and reduce transmission within households and communities.

## Author Contributions

Ana Beatriz Nardelli da Silva contributed to the research, writing, and editing (joint first authorship). Giovanna Lourenço Cei contributed to writing, and Adriani Ferreira do Carmo provided dermatological expertise and corrections. Arlisson Macedo Rodrigues and Maitê Silva Martins Gadelha contributed to overall guidance, supervision, and editing.

## Funding

This research was supported by the British Council through the International Science Partnerships Fund (ISPF), Application No. 1649. The authors gratefully acknowledge this support, which has been instrumental in facilitating the development of this work.

## Disclosure

All authors reviewed, edited, and approved the final manuscript prior to submission.

## Ethics Statement

Ethical approval was obtained from the Institutional Ethics Committee of the João de Barros Barreto University Hospital where the case was conducted. The study was conducted in accordance with applicable ethical guidelines for case reports.

## Consent

Written informed consent for publication of this case report and accompanying images was obtained directly from the patient. The patient was informed about the nature of the publication and agreed to the use of anonymized clinical data and photographs.

## Conflicts of Interest

The authors declare no conflicts of interest.

## Patient Perspective

The patient expressed satisfaction with the final diagnosis and treatment received, noting that previous therapies had not provided relief. He also highlighted the impact of the illness on his daily life and well‐being and reported feeling reassured by the resolution of symptoms and recovery at discharge.

## Supporting Information

Additional supporting information can be found online in the Supporting Information section.

## Supporting information


**Supporting Information** CARE checklist.

## Data Availability

The data that support the findings of this study are available on request from the corresponding author. The data are not publicly available due to privacy or ethical restrictions.
