# Supplementary material for: Sepsis Secondary to Crusted Scabies Following Iatrogenic Treatment for Hansen’s Disease in an Indigenous Amazonian Patient: A Case Report
Source: Case Rep Infect Dis. 2026 Sep 2;2026:5279255. doi: 10.1155/crdi/5279255 (PMC13536483; doi:10.1155/crdi/5279255)
Supplement: Supplementary file 1 [file CRDI-2026-5279255-s001.docx]

**CARE  Checklist  of  information  to  include  when  writing  a  case  report****Topic Item Checklist item description Reported on Line**

**Title**

**1** The diagnosis or intervention of primary focus followed by the words “case report” (**PAGE 1)**

**Key Words**

**2** 2 to 5 key words that identify diagnoses or interventions in this case report, including "case report" (**PAGE 2)**

**Abstract (no references)**

**_3a_**

Introduction:

What is unique about this case and what does it add to the scientific literature? (**PAGE 2)**

**3b** Main symptoms and/or important clinical findings (**PAGE 2)**

**3c** The main diagnoses, therapeutic interventions, and outcome (**PAGE 2)**

**3d** Conclusion:

What is the main “take-away” lesson(s) from this case? (**PAGE 2)**

**Introduction**

**4** One or two paragraphs summarizing why this case is unique (**may include references**)

(**PAGE 3)**

**Patient Information**

**5a** De-identified patient specific information (**PAGE 3)**

**5b** Primary concerns and symptoms of the patient. (**PAGE 3)**

**5c** Medical, family, and psycho-social history including relevant genetic information (**PAGE 3)**

**5d** Relevant past interventions with outcomes (**PAGE 3)**

**Clinical Findings Timeline**

**Diagnostic**

**Assessment**

**Therapeutic**

**Intervention**

**Follow-up and Outcomes**

**6** Describe significant physical examination (PE) and important clinical findings (**PAGE 3,4)**

**^7^** Historical and current information from this episode of care organized as a timeline (**PAGE 4)**.

**8a** Diagnostic testing (such as PE, laboratory testing, imaging, surveys) (**PAGE 4)**

**8b** Diagnostic challenges (such as access to testing, financial, or cultural) (**PAGE 4)**

**8c** Diagnosis (including other diagnoses considered) (**PAGE 3)**

**8d** Prognosis (such as staging in oncology) where applicable (**NOT** **APPLICABLE**)

**9a** Types of therapeutic intervention (such as pharmacologic, surgical, preventive, self-care) (**PAGE 3,4)**

**9b** Administration of therapeutic intervention (such as dosage, strength, duration)(**PAGE 4)**

**9c** Changes in therapeutic intervention (with rationale) (**PAGE 3,4)**

**10a** Clinician and patient-assessed outcomes (**PAGE 3,4)**

**10b** Important follow-up diagnostic and other test results (**PAGE 3,4,5)**

**10c** Intervention adherence and tolerability (How was this assessed?) (**PAGE 4)**

**10d** Adverse and unanticipated events (**PAGE 4)**

**Discussion**

**11a** A scientific discussion of the strengths AND limitations associated with this case report (**PAGE 7)**

**11b** Discussion of the relevant medical literature **with references** (**PAGE 7)**

**11c** The scientific rationale for any conclusions (including assessment of possible causes) (**PAGE 7)**

**11d** The primary “take-away” lessons of this case report (without references) in a one paragraph conclusion (**PAGE 7)**

**Patient Perspective**

**12** The patient should share their perspective in one to two paragraphs on the treatment(s) they received (**PAGE 8)**

**Informed Consent 13** Did the patient give informed consent? Please provide if requested (**PAGE 8)** **Yes**
